# Discovering a trans-omics biomarker signature that predisposes high risk diabetic patients to diabetic kidney disease

**DOI:** 10.1038/s41746-022-00713-7

**Published:** 2022-11-02

**Authors:** I-Wen Wu, Tsung-Hsien Tsai, Chi-Jen Lo, Yi-Ju Chou, Chi-Hsiao Yeh, Yun-Hsuan Chan, Jun-Hong Chen, Paul Wei-Che Hsu, Heng-Chih Pan, Heng-Jung Hsu, Chun-Yu Chen, Chin-Chan Lee, Yu-Chiau Shyu, Chih-Lang Lin, Mei-Ling Cheng, Chi-Chun Lai, Huey-Kang Sytwu, Ting-Fen Tsai

**Affiliations:** 1 Department of Nephrology, Chang Gung Memorial Hospital, Keelung, 204 Taiwan; 2 Community Medicine Research Center, Chang Gung Memorial Hospital, Keelung, 204 Taiwan; 3 College of Medicine, Chang Gung University, Taoyuan, 333 Taiwan; 4 Advanced Tech BU, Acer Inc., New Taipei City, 221 Taiwan; 5 Metabolomics Core Laboratory, Healthy Aging Research Center, Chang Gung University, Taoyuan, 333 Taiwan; 6 Institute of Molecular and Genomic Medicine, National Health Research Institutes, Miaoli, 350 Taiwan; 7 Department of Thoracic and Cardiovascular Surgery, Chang Gung Memorial Hospital, Linkou Taoyuan, 333 Taiwan; 8 Department of Nursing, Chang Gung University of Science and Technology, Taoyuan, 333 Taiwan; 9 Department of Gastroenterology and Hepatology, Chang Gung Memorial Hospital, Keelung, 204 Taiwan; 10 Clinical Metabolomics Core Laboratory, Chang Gung Memorial Hospital, Linkou Taoyuan, 333 Taiwan; 11 Department of Biomedical Sciences, College of Medicine, Chang Gung University, Taoyuan, 333 Taiwan; 12 Department of Ophthalmology, Chang Gung Memorial Hospital, Keelung, 204 Taiwan; 13 National Institute of Infectious Diseases and Vaccinology, National Health Research Institutes, Miaoli, 350 Taiwan; 14 Department & Graduate Institute of Microbiology and Immunology, National Defense Medical Center, Taipei, 114 Taiwan; 15 Department of Life Sciences and Institute of Genome Sciences, National Yang Ming Chiao Tung University, Taipei, 112 Taiwan; 16 Center for Healthy Longevity and Aging Sciences, National Yang Ming Chiao Tung University, Taipei, 112 Taiwan

**Keywords:** Kidney, Genome-wide association studies, Metabolism

## Abstract

Diabetic kidney disease is the leading cause of end-stage kidney disease worldwide; however, the integration of high-dimensional trans-omics data to predict this diabetic complication is rare. We develop artificial intelligence (AI)-assisted models using machine learning algorithms to identify a biomarker signature that predisposes high risk patients with diabetes mellitus (DM) to diabetic kidney disease based on clinical information, untargeted metabolomics, targeted lipidomics and genome-wide single nucleotide polymorphism (SNP) datasets. This involves 618 individuals who are split into training and testing cohorts of 557 and 61 subjects, respectively. Three models are developed. In model 1, the top 20 features selected by AI give an accuracy rate of 0.83 and an area under curve (AUC) of 0.89 when differentiating DM and non-DM individuals. In model 2, among DM patients, a biomarker signature of 10 AI-selected features gives an accuracy rate of 0.70 and an AUC of 0.76 when identifying subjects at high risk of renal impairment. In model 3, among non-DM patients, a biomarker signature of 25 AI-selected features gives an accuracy rate of 0.82 and an AUC of 0.76 when pinpointing subjects at high risk of chronic kidney disease. In addition, the performance of the three models is rigorously verified using an independent validation cohort. Intriguingly, analysis of the protein–protein interaction network of the genes containing the identified SNPs (RPTOR, CLPTM1L, ALDH1L1, LY6D, PCDH9, B3GNTL1, CDS1, ADCYAP and FAM53A) reveals that, at the molecular level, there seems to be interconnected factors that have an effect on the progression of renal impairment among DM patients. In conclusion, our findings reveal the potential of employing machine learning algorithms to augment traditional methods and our findings suggest what molecular mechanisms may underlie the complex interaction between DM and chronic kidney disease. Moreover, the development of our AI-assisted models will improve precision when diagnosing renal impairment in predisposed patients, both DM and non-DM. Finally, a large prospective cohort study is needed to validate the clinical utility and mechanistic implications of these biomarker signatures.

## Introduction

Diabetes mellitus (DM) remains a major medical challenge and affects 463 million adults globally^1,2^. Diabetic kidney disease (DKD) is the leading cause of end-stage kidney disease worldwide^3,4^. These conditions are strongly associated with high rates of cardiovascular disease and mortality^5,6^, as well as very high medical expenditure^7^. DKD risk prediction via reliable biomarkers is currently an unmet clinical need. Urinary albumin excretion and serum creatinine are the common clinical biomarkers used for diagnosis and staging of renal impairment among chronic kidney disease (CKD) patients. However, abnormalities in these two parameters often indicate existing kidney damage rather than a predisposition of renal impairment in the future^8^. Their usefulness when estimating glomerular filtration rate (eGFR) is subject to several limitations due to various potential confounders, such as age, sex, muscle mass, and changes in glomerular hemodynamics due to hyperfiltration that is secondary to the patient’s hyperglycaemia status^9^. Other biomarkers of kidney injury, including cystatin C, kidney injury molecule 1, neutrophil gelatinase-associated lipocalin, and liver fatty acid-binding protein, are not specific to DKD and also have disadvantages. Furthermore, liver or thyroid dysfunction, an alteration in urine volume, a change in creatinine concentration, and treatment with various medications can also affect the levels of these biomarkers^10–13^. An approach that accurately predisposes high risk DM patients to DKD remains urgently needed.

High-throughput omics approaches have revolutionized biomarker research and have helped to advance our understanding of renal progression in DM patients^14^. Given the various hemodynamic and metabolic disarrangements exerted by hyperglycaemia on kidney tissue, metabolomic analysis represents a very useful way of addressing this clinical issue^15^. In addition, genome-wide association studies (GWAS) have been able to identify a number of potential genes, loci, and single-nucleotide polymorphisms (SNPs) that are associated with DKD, which implies that genetic susceptibility is part of the pathogenesis of DKD^16–18^. However, up to the present, the development of biomarkers focusing on the genomic-metabolomic signatures specific to DKD has not taken place.

To fill this knowledge gap, we conduct a trans-omics study that integrated high dimensional data collected from an extensive clinical information dataset, an untargeted metabolomics dataset and a lipidomics dataset, as well as genome-wide SNP genotyping. We adopt a machine learning (ML) methodology to delineate the complex biological processes associated with four health conditions: control subjects, subjects with DM, subjects with CKD and subjects with DKD. Furthermore, we performed protein connectivity mapping of the genes containing the relevant SNPs in order to determine their potential roles in the molecular pathogenesis of CKD and DKD, specifically their functional connectivity and their protein–protein interaction network.

## Results

### Clinical characteristics

The 618 subjects were split into training and testing cohorts with 557 and 61 subjects, respectively. The baseline characteristics of the subjects are presented in Supplementary Table 1. Of the subjects, 338 subjects (54.7%) were controls, 106 (17.2%) had type 2 DM, 73 (11.8%) had non-diabetic CKD, and 101 subjects (16.3%) had DKD. The mean age of the study population was 63.8 ± 12.9 years old and included 287 males (46.4%). The median eGFR was 83.0 mL/min/1.73 m^2^. The DKD patients were more like to be older, to have hypertension, to have a higher serum triglyceride level, a higher level of calcium, and a higher level of insulin (Table 1). The external validation cohort, which is independent of the training cohort, consisted of 178 subjects (control 100, DM 26, non-diabetic CKD 22, and DKD 30) with a mean age of 60.6 years old and a mean eGFR of 84.1 mL/min/1.73 m^2^ (Supplementary Table 1).

**Table 1:** Baseline characteristics of study population stratified by groups.

	Normal control	Diabetes	Non-diabetic CKD	Diabetic kidney disease	*P* value
Parameters	*n* = 338	*n* = 106	*n* = 73	*n* = 101	
Age, years	60.4 ± 13.2	66.1 ± 10.9	68.6 ± 12.8	69.5 ± 9.5	<0.001
Male, No. (%)	153 (45.3%)	52 (49.1%)	29 (39.7%)	53 (52.5%)	0.35
Comorbidities					
Hypertension, No. (%)	87 (25.7%)	71 (67.0%)	41 (56.2%)	69 (68.3%)	<0.001
Obesity, No. (%)	173 (51.2%)	105 (99.1%)	64 (87.7%)	99 (98.0%)	<0.001
Personal habits					
Smoking, No. (%)	94 (27.8%)	30 (28.3%)	17 (23.3%)	26 (25.7%)	0.851
Alcohol drinking, No. (%)	151 (44.7%)	28 (26.4%)	23 (31.5%)	14 (13.9%)	0.002
Anthropometrics					
Body mass index, kg/m2	26.1 ± 4.1	27.5 ± 4.1	27.2 ± 4.0	27.3 ± 4.0	0.003
Systolic BP, mmHg	129.8 ± 16.4	135.4 ± 16.4	142.1 ± 16.4	139.0 ± 18.4	<0.001
Diastolic BP, mmHg	76.8 ± 11.1	78.2 ± 9.8	81.6 ± 12.5	77.7 ± 10.3	0.059
Laboratory					
eGFR, mL/min per 1.73 m2	89.8 ± 17.3	89.5 ± 20.6	66.9 ± 24.9	63.0 ± 22.1	<0.001
BUN, mg/dL	14.7 (7.1, 39.2)	15.4 (5.8, 33.1)	17.6 (8.4, 54.6)	19.0 (6.3, 75.4)	<0.001
Serum creatinine, mg/dL	0.8 (0.4, 1.2)	0.8 (0.4, 1.2)	1.0 (0.5, 2.0)	1.0 (0.6, 6.9)	<0.001
Serum albumin, mg/dL	4.6 (3.1, 5.5)	4.5 (3.7, 246.4)	4.7 (4.0, 5.5)	4.5 (3.5, 17.9)	0.913
Cholesterol, mg/dL	195 (99, 377)	166 (102, 262)	188 (96, 323)	173 (92, 339)	<0.001
Triglycerides, mg/dL	106 (25, 457)	134 (43, 523)	134 (45, 433)	139 (52, 1225)	<0.001
hs-C reactive protein, mg/L	1.1 (0.2, 46.7)	1.1 (0.2, 73.1)	1.1 (0.2, 67.4)	1.4 (0.1, 59.6)	0.036
Urine albumin/creatinine ratio, mg/g	6.0 (1.3, 28.2)	10.0 (2.4, 28.4)	66.5 (3.0, 3590.4)	51.5 (2.6, 3792.1)	<0.001
Vitamin D, ug/mL	584.0 (179.8, 3442.0)	549.8 (114.0, 3266.0)	551.1 (119.0, 3374.0)	580.2 (22.3, 3295.0)	0.140
Intact parathyroid hormone, pmol/L	42.3 (6.0, 122.5)	39.8 (15.8, 121.0)	48.6 (20.5, 124.3)	38.1 (11.9, 199.0)	0.362
Serum calcium, mg/dL	9.3 (6.6, 10.3)	9.4 (8.1, 10.2)	9.5 (8.5, 10.2)	9.6 (8.0, 10.5)	<0.001
Serum phosphate, mg/dL	3.6 (2.1, 5.7)	3.5 (2.1, 5.1)	3.6 (2.2, 4.8)	3.6 (2.1, 5.3)	0.407
Insulin, uU/mL	9.8 (0.5, 47.1)	11.9 (2.2, 84.9)	11.5 (4.7, 49.7)	14.0 (1.2, 84.3)	<0.001
LDL-C / HDL-C, mg/dL	2.4 (0.7, 5.8)	2.2 (0.7, 5.1)	2.3 (0.8, 5.9)	2.2 (0.6, 5.1)	0.225
Urine urea, mg/dL	856.5 (359.7, 1923.3)	836.2 (343.5, 1762.7)	817.2 (281.7, 1612.0)	694.7 (117.6, 1685.9)	<0.001
Glycated Hemoglobin, %	5.7 (4.5, 6.4)	6.7 (5.3, 10.2)	5.9 (4.6, 6.4)	6.9 (5.2, 14.4)	<0.001
Glucose, mg/dL	96 (76, 125)	126 (84, 252)	100 (83, 124)	131 (69, 400)	<0.001

The values are expressed as means ± SD or median (Min, Max) or n (%).

*CKD* chronic kidney disease, *BUN* blood urea nitrogen, *eGFR* estimated glomerular filtration rate, *hs-C reactive protein* high-sensitivity C reactive protein, *LDL-C/HDL-C* low density lipoprotein-cholesterol/high density lipoprotein-cholesterol.

The *p* value was performed by *F* test in ANOVA and Chi-square test for comparison of the four groups.

### Artificial intelligence (AI)-assisted identification of multi-omics signatures: discovery and validation

**Fig. 1:**
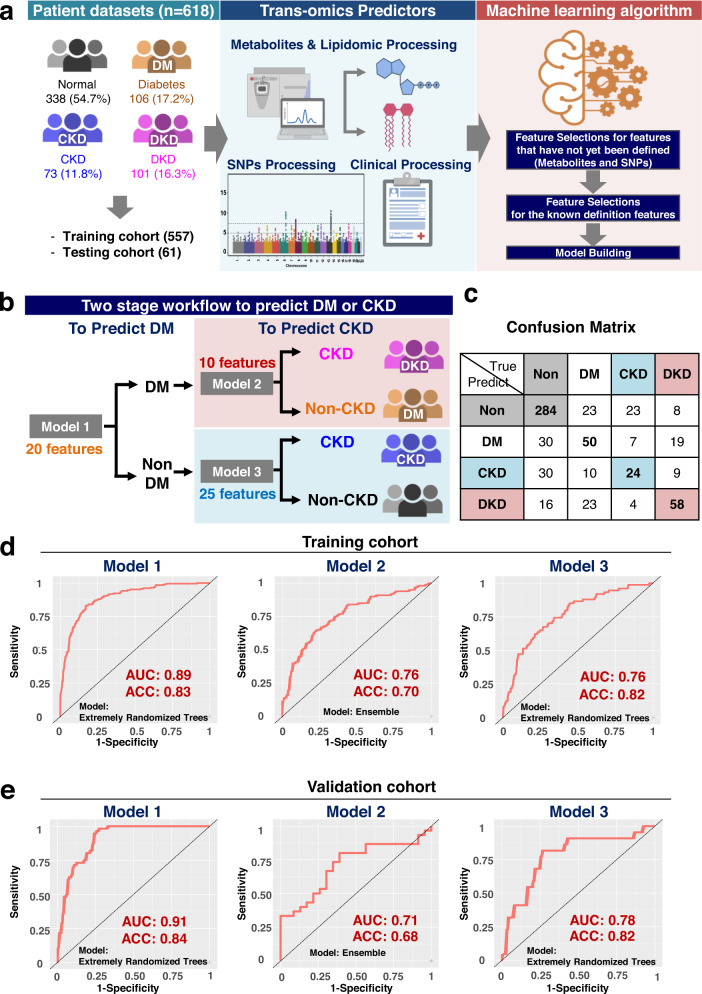
Study flow chart, machine learning algorithms and their performance when using the three prediction models. **a** The scheme illustrates the data processing and machine learning workflow that integrates the non-targeted metabolites, lipidomics (P180-metabolites), SNPs, and clinical data. **b** The two stage modeling workflow used to predict diabetes mellitus (DM) and chronic kidney disease (CKD). **c** The Confusion Matrix of prediction accuracy obtained by aggregating the three models into four groups. For example, the Non DM and Non CKD predicted label is predicted as Non DM in model 1 and predicted to be Non CKD in model 3. **d**–**e** The receiver operating characteristic (ROC) curves of Model 1, Model 2 and Model 3 that were used for predicting DM and CKD in the training cohort (**d**) and the validation cohort (**e**). Abbreviations include DM diabetes, CKD chronic kidney diseases, DKD diabetic kidney diseases, AUC area under the curve, ACC accuracy, SVM Support Vector Machine. The figure was created with BioRender.com.

To categorize the biomarkers associated with DM and CKD, we carried out an AI-based study that integrated three types of features (metabolomics, SNPs and clinical information). The analysis consisted of three parts: (1) an analysis of the performance of metabolomics, SNPs and the clinical information data associated with the four groups of subjects; (2) feature selection; and (3) model derivation and validation (Fig. 1a and Supplementary Fig. 1).

We used the identified defined features from the feature importance list to derive the three models. Model 1 selected 20 features that differentiated DM (Supplementary Table 2); Model 2 selected 10 features that identified DKD (DM patients with CKD) (Supplementary Table 3); Model 3 selected 25 features that distinguished CKD in non-DM patients (Supplementary Table 4). To carry out model derivation and validation, the subjects were randomly assigned into 10 sets in order to carry out a 10-fold cross validation as part of the second stage of model building (Fig. 1b). To evaluate the performance of the three models, Area Under Curve (AUC) and accuracy rate were used. Of the five ML models tested in Model 1 (Fig. 1b), the extremely randomized trees (extra-tree) model gave the best performance in terms of AUC and accuracy rate (Supplementary Fig. 2a). The top 20 features selected by AI gave the performance with an accuracy rate of 0.83 and an AUC of 0.89 when used to differentiate DM and non-DM (Supplementary Table 5 and Supplementary Fig. 2a). In Model 2 (Fig. 1b), the Ensemble model gave the best performance. The top 10 features selected by AI gave the performance with an accuracy rate of 0.70 and an AUC of 0.76 when used to differentiate CKD and non-CKD among DM patients (Supplementary Table 6 and Supplementary Fig. 2b). In Model 3 (Fig. 1b), the Extra-Tree model gave the best performance. The top 25 features selected by AI gave the performance with an accuracy rate of 0.82 and an AUC of 0.76 when used to differentiate CKD and non-CKD among non-DM patients (Supplementary Table 7 and Supplementary Fig. 2c). The confusion matrix of the accuracy is summarized in Fig. 1c. The Receiver Operating Characteristic (ROC) analysis of the 10-fold cross validation for Model 1 (Extra Tree; AUC 0.89), Model 2 (Ensemble; AUC 0.76), Model 3 (Extra Tree; AUC 0.76) are presented in Fig. 1d. Furthermore, we used an external validation cohort that had been collected independently during 2019 and 2020 to carry out a rigorous validation of the performance of the three models. Consistently, the various analyses of the validation cohort revealed a similar result to that obtained using the training cohort (Fig. 1e**;** Supplementary Table 8).

### A biomarker signature that identifies high-risk subjects among DM patients who are predisposed to renal impairment

**Fig. 2:**
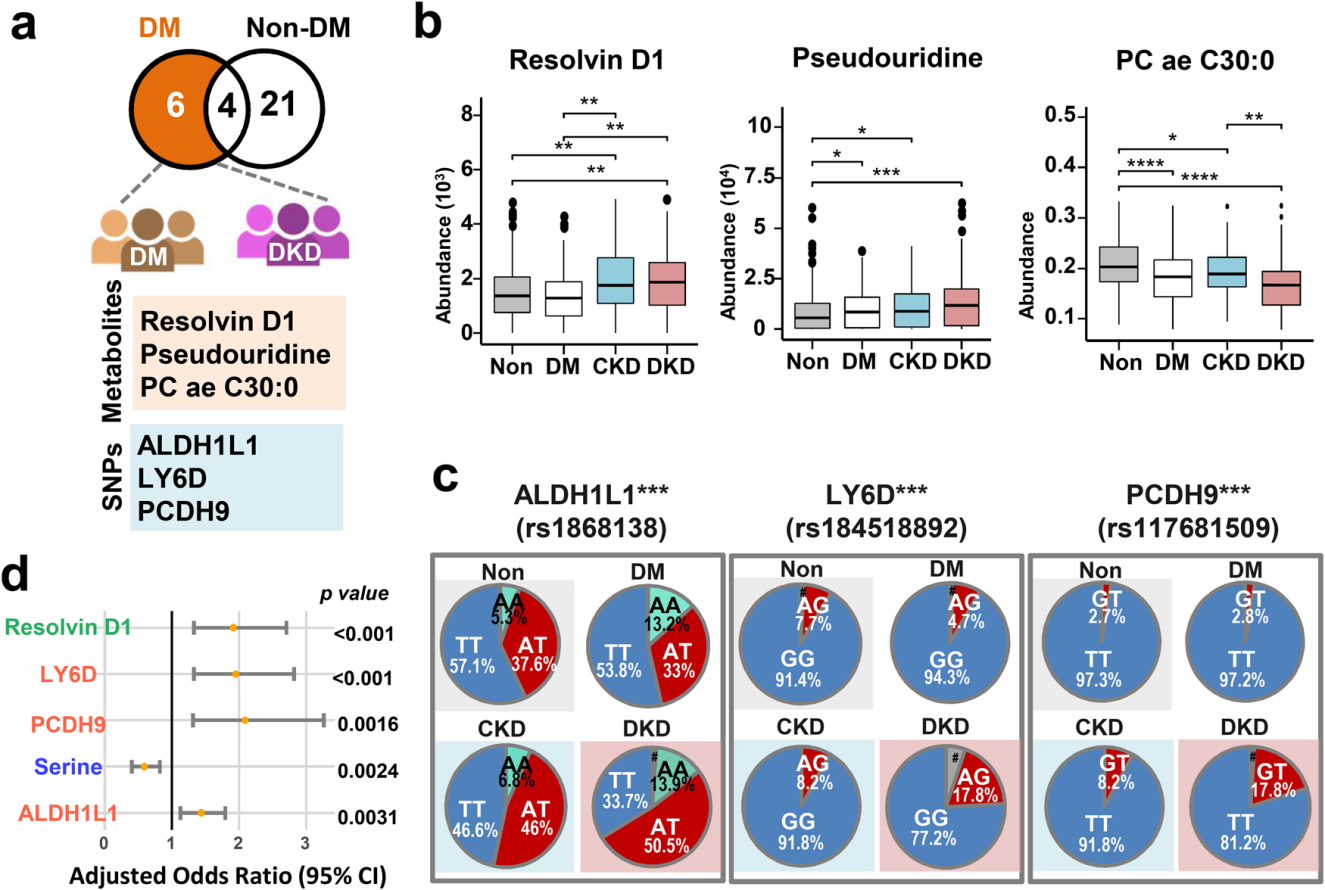
Selected features for identifying renal dysfunction in DM patients, namely DKD (Model 2). **a** Venn diagram of AI-selected features in Model 2. **b** Box plot of AI-selected features of metabolites in Model 2. The statistical analysis with p-values was performed by ANOVA for significant metabolites in the four groups. The t-test was used for multiple comparisons within the four groups test. Box plot: Box plot includes a box and a set of whiskers. The lower line of the box is represented as Q1 (25th percentile). The upper line of the box is represented as Q3 (75th percentile). The middle and bold line in the box is represented as median. In general, the boundary of the lower and upper whiskers is 1.5 interquartile ranges (IQR, IQR = Q3 − Q1) below the Q1 and 1.5 IQR above the Q3. The extreme values outside this boundary are considered as outliers and plotted as black dots. If all data points are between Q1 − 1.5 x IQR and Q3 + 1.5 x IQR, the boundary of the lower and upper whiskers should be minimum and maximum of the data. The error bars in this figure represent the lower and the upper whiskers defined above. **c** Pie charts indicating the genotype frequencies of SNPs using the SNP datasets obtained from the subjects. Number sign indicates that the signal from the SNP array was lower than the calling rate. The χ2 test was used for comparisons of genotype frequencies within the four groups. **d** Adjusted odds ratios of factors in backward logistic regression procedure associated with the occurrence of CKD among DM patients. The Wald test was used to construct 95% confidence interval (CI) and test the significance of adjusted odds ratios of risk factors. The error bars represent the lower bound and the upper bound of adjusted odds ratio of 95% confidence interval. *p < *0.05*, **p < *0.01*, ***p < *0.001*, ****p < *0.0001*. ALDH1L1 aldehyde dehydrogenase 1 family member L1, LY6D lymphocyte antigen 6 family member D, PCDH9 protocadherin 9.

Among all the biomarkers that are potentially significant when pinpointing the occurrence of DKD among DM patients (Model 2), six features were selected by AI from all of the groups, namely three metabolites and three annotated SNPs (Fig. 2a). The three metabolites include one bioactive lipid mediator (resolvin D1), a purine and pyrimidine metabolite (pseudouridine), and one phospholipid (phosphatidylcholine C-30:0). Notably, the abundances of resolvin D1 and of pseudouridine were significantly higher in the DKD patients compared to the controls; by way of contrast, the levels of the three phospholipids were significantly lower in the DKD patients compared to the controls (Fig. 2b). Among the three SNPs selected, two of them (rs1868138 and rs117681509) are intron variants located within the ALDH1L1 and PCDH9 genes, respectively; the other (rs184518892) is a synonymous variant of the LY6D gene (Fig. 2c; Supplementary Table 3). When these protein-coding genes were examined, we found that the frequencies of AT genotype of the ALDH1L1 gene (rs1868138), the AG genotype of LY6D gene (rs184518892) and the GT genotype of PCDH9 gene (rs117681509) were significantly higher among DKD patients compared to DM patients (*p* < 0.001; Chi-square test; Fig. 2c). Notably, expression of all three of these protein-coding genes is able to be detected in various major organs associated with DKD, namely the kidneys, pancreas, liver, adipose tissue, and heart^19^ (Supplementary Tables 9 and 10).

We conducted logistic regression analysis to further elucidate the relationships between the AI-selected biomarkers and the different disease groups. It was found that an increase in the adjusted odds ratios (OR) was present for the occurrence of DKD in relation to resolvin D1 (*p* < 0.001; Wald test), the PCDH9 genotype (*p* = 0.0016; Wald test), the LY6D genotype (*p* < 0.001; Wald test), and the ALDH1L1 genotype (*p* = 0.0031; Wald test). Conversely, a higher level of serine (*p* = 0.0024; Wald test) appeared to be associated with a lower risk of the occurrence of DKD (Fig. 2d). It should be noted that ALDH1L1 is highly expressed in the kidneys, which supports the possibility that this gene may play a role in the pathogenesis of DKD.

### A biomarker signature that identifies subjects at high risk of CKD among non-DM patients

Twenty-one features were selected by AI as being associated with the renal impairment among non-diabetic patients; these were age, body mass index, 15 metabolites, and five annotated SNPs (Model 3; Supplementary Fig. 3a–c). The five SNPs selected by AI are all located within protein-coding genes (Supplementary Table 4). Interestingly, four of the above-mentioned genes, B3GNTL1, CDS1, FAM53A and ADCYAP1, are expressed at significant levels in the kidney^19^, which supports the hypothesis that they might play roles in kidney pathogenesis. Specifically, significantly higher frequencies of the TT genotype of B3GNTL1, the CT genotype of CDS1, the AT genotype of CCDC182, and the CT genotype of FAM53A were found in the non-diabetic CKD patients compared to the other groups (*p* < 0.001; χ2 test). Conversely, a significant decrease in the frequency of the AG genotype of ADCYAP1 gene was found among non-diabetics CKD patients compared to the other groups (*p* < 0.001; Chi-square test; Supplementary Fig. 3d).

Logistic regression analysis revealed that there was a significant increase in the risk association of non-diabetic CKD patients with the following five features: age (*p* < 0.001; Wald test), abundance of mannose/inositolI (*p* < 0.001; Wald test), the FAM53A genotype (*p* = 0.0026; Wald test), the CDS1 genotype (*p* = 0.0065; Wald test), and the CCDC182 genotype (*p* = 0.0071; Wald test). On the other hand, there was a significant decrease in the risk association of non-diabetic CKD with the B3GNTL1 genotype (*p* = 0.0012; Wald test), and the abundance of PC C30:2 (*p* = 0.0073; Wald test) (Supplementary Fig. 3e).

### The protein–protein interaction network of the genes containing the AI-selected SNPs reveals linkage between DM and CKD

**Fig. 3:**
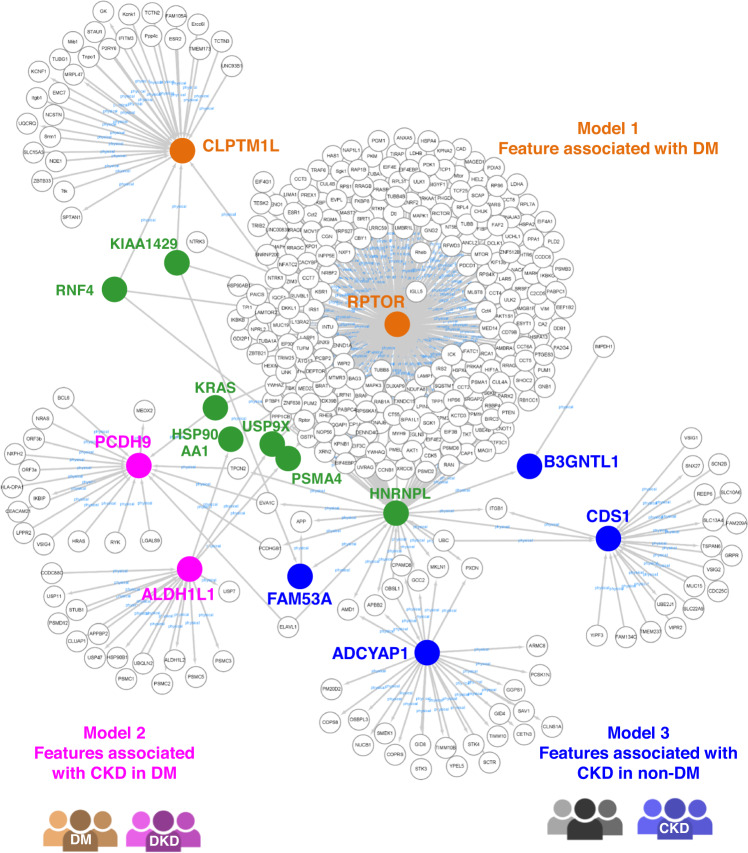
The protein–protein interaction network of the genes containing the AI-assisted identified SNPs. The protein-coding genes containing the SNPs identified by AI were used to build up a protein–protein interaction network. Ranking of the protein-coding genes in the feature importance of the three models: Model 1, RAPTOR (rank 23) and CLPTM1L (rank 25); Model 2, PCDH9 (rank 9) and ALDH1L1 (rank 7); Model 3, FAM53A (rank 19), ADCYAP (rank 16), B3GNTL1 (rank 10) and CDS1 (rank 13). The figure was created using the open-source software Cytoscape. CLPTM1L cleft lip and palate transmembrane protein 1-like, RPTOR regulatory associated protein of mTOR complex 1, ALDH1L1 aldehyde dehydrogenase 1 family member L1, PCDH9 protocadherin 9, B3GNTL1 UDP-GlcNAc:BetaGal Beta-1,3-N-Acetylglucosaminyltransferase like 1, CDS1 CDP-Diacylglycerol synthase 1, FAM53A family with sequence similarity 53 member A, ADCYAP1 adenylate cyclase activating polypeptide 1, RNF4 ring finger protein 4, USP9X, ubiquitin specific peptidase 9 X-linked, HSP90AA1 heat shock protein 90 alpha family class a member 1, PSMA4 proteasome 20 S subunit alpha 4, HNRNPL heterogeneous nuclear ribonucleoprotein L.

We conducted a protein–protein interaction network analysis of the various genes identified by Model 1, Model 2 and Model 3 (Supplementary Tables 9 and 10) using the BioGRID database^20^. We found that RPTOR, which was selected in Model 1 as identifying the occurrence of DM, is the main hub gene that connects the various protein-coding genes selected in Model 2 (identification of DKD), and Model 3 (identification of CKD). Eight of the protein-coding genes identified in Model 1 (RAPTOR and CLPTM1L), Model 2 (PCDH9 and ALDH1L1), and Model 3 (FAM53A, ADCYAP, B3GNTL1 and CDS1) are able to be connected to each other and they form an obvious protein–protein interaction network (Fig. 3). In addition, it can be seen that the HNRNPL protein also acts as a hub and this gene connects the four proteins identified in Model 3 with RPTOR (Fig. 3). This overall interaction network suggests that, at the molecular level, there seems to be an overlap between the pathogenesis of DM and the pathogenesis of CKD, and that these various interconnected factors might be able to affect the progression of renal impairment among DM patients.

## Discussion

The disease burden of DKD creates a tremendous worldwide load both at the personal and at the national level. However, current diagnostic methods are insufficient to provide good risk stratification and have failed to reveal the various molecular pathways that underlie disease in CKD or DM patients. Here we have applied AI-based ML-algorithms to carry out vigorous feature selection and to build three models; these are able to differentiate DM and non-DM individuals (mode 1, Extra Tree; AUC 0.89), CKD and non-CKD in DM patients (model 2, Ensemble; AUC 0.76), and CKD and non-CKD in non-DM patients (model 3, Extra Tree; AUC 0.76) with good prediction performance. Additionally, we identified a biomarker signature made up of several phospholipids (Phosphatidylcholine and Lysophosphatidylcholine), a lipid mediator (resolvin D1) and a purine/pyrimidine metabolism mediator (pseudouridine) that are associated with DKD. Furthermore, the findings related to specific genotypes of the annotated SNPs associated with CKD or DKD highlight that there seems to be genetic susceptibility in predisposed subjects toward renal impairment. Intriguingly, analysis of the protein–protein interaction network of the genes containing the SNPs identified by the three models revealed that, at the molecular level, there seems to be an overlap between the pathogenesis of DM and the pathogenesis of CKD; it also identified various interconnected factors that may affect the progression of renal impairment among DM patients, namely the occurrence of DKD.

The ability to generate trans-omics big data makes machine learning approaches suitable for obtaining both biological and translational perspectives related to questions in kidney biology^21^. These methodologies have been able to identify altered lipidomic profiles associated with the risk of renal progression among CKD patients^22^ and to uncover proteomics and metabolomics biomarkers associated with early *vs*. advanced stage DKD^23^. Here, we have utilized AI-based techniques combined with traditional approaches to select subsets of genomic and metabolomic features that give the best performances and avoided overfitting the training data^24^. Importantly, the performances of the three models built in this study were verified using an independent validation cohort and this cohort gave similar results that are comparable to those obtained from the training cohort. Our findings indicate that an AI-assisted approach is able to considerably improve the precision diagnosis of CKD and DKD and this represents an important tool for providing insights into the potential molecular mechanism(s) that underlies various renal disease states.

The use of serum metabolites increases accuracy when estimating kidney function during DKD compared to serum proteins^23^. Our findings are consistent with previous reports whereby there are perturbations affecting the metabolites associated with glycerol-lipid metabolsm and oxidative stress^25^, such as glycerol-3-galactoside^23^ and asymmetric dimethylarginine^26^, as well as purine and pyrimidine metabolism, namely pseudouridine^27^, in patients with DKD^28^. Diacylglycerol can activate protein kinase C, which, in turn, affects various inflammatory and growth-promoting pathways^9^. Conversely, serine, glutamine and lysophosphatidylcholine C18:2 are associated with a decreased risk of DM^29^. We also found an increase of serum kynurenine levels in CKD patients. Kynurenine is derived from tryptophan metabolism; it appears to play a role in several biological processes, including energy metabolism, the pro-inflammatory response, atherosclerosis and oxidative stress^30,31^. Increased concentrations of kynurenine have been found to be associated with acute kidney injury, CKD, DKD and cardiovascular diseases^32,33^. Intriguingly, we also found an increase in the level of resolvin D1 in DKD patients; this molecule is a bioactive lipid mediator derived from omega-3 fatty acid. Previous studies have shown that resolvin D1 seems to be able to promote the resolution of the inflammatory response and has anti-oxidant effects^34,35^. Accordingly, it is possible that the increase in resolvin D1 is a compensatory effect in response to the elevated inflammation and increased oxidative stress in DKD patients. Additionally, some of the biomarkers discovered in the present study may serve as mediators of the post-transcriptional and/or post-translational modifications involved in progression of injury-repair processes in the kidney under hyperglycemia.

Genomics studies have identified several genetic variants associated with kidney disease; however, their functional effects remain poorly understood^36^. Previous publications have reported a variety of SNPs that are associated with DKD, including CNDP1, FRMD3, RGMA-MCTP2 (rs12437854), AFF3 (rs7583877), ERBB4 (rs7588550), COL4A3 (rs55703767), and MUC7^37,38^. However, combination of SNPs does not improve discrimination of DKD or CKD beyond traditional risk factors in a range of ethnic populations^39–41^. In this study, several protein-coding genes containing SNPs were selected by AI and used as important features when distinguishing the various groups; this highlights the importance of genetic susceptibility to renal dysfunction among predisposed subjects. Among the SNPs associated with DKD, ALDH1L1 encodes a protein that belongs to the aldehyde dehydrogenase family and is associated with the NADPH pathway. Furthermore, LY6D acts as a specification marker during the earliest stage specification of lymphocytes during B-cell and T-cell development. In addition, PCDH9 is responsible for encoding a potential calcium-dependent cell-adhesion protein. On the other hand, B3GNTL1, CDS1, ADCYAP1 and FAM53A SNPs are associated with CKD among non-DM patients. Among these, B3GNTL1 is a putative glycosyltransferase, CDS1 catalyzes the conversion of phosphatidic acid to diacylglycerol and ADCYAP1 encodes a secreted protein that is involved in the control of glucose homeostasis and the regulation of insulin secretion in pancreatic beta cells^42^. Intercommunication between these genetic factors, which was revealed by the connectivity map, deserves further functional investigation in order to decipher their roles in the pathogenesis of DM and CKD.

There are several limitations to the present study in spite of the meticulous application by us of a range of ML approaches to leverage the big datasets associated with our multi-omics data in order to gain insights into disease phenotype association. Firstly, we used the levels of circulating biomarkers to identify disease association. Analysis of multiple tissues, for example, kidney, liver, and muscle, needs to be performed to clarify the sources of these biomarkers. Secondly, renal biopsy was not conducted to ascertain the histopathological definition of DKD in a community setting due to its intrusiveness. Nevertheless, various clinical clues that are indicative of superimposed glomerulonephritis, such as hematuria, red cell cast or nephrotic range proteinuria, were minimal among our participants. Thirdly, this is a cross-sectional study derived from a single ethnic group. Although an external validation cohort was used, our findings need to be verified on a larger scale and using a number of ethnically diverse cohorts. Fourthly, questions relating to the cause-and-effect relationship between the genes identified in the present study need to be investigated using transcriptional profiling, a cell platform system and/or an animal model approach. Finally, longitudinal studies are needed to validate the usefulness of our models when distinguishing renal progression; this is essential so that the trans-omics signature can become a biomarker profile that can be used for personalized medicine on DKD patients.

In conclusion, our findings reveal the potential of employing ML models to augment traditional methods and to help to identify molecular mechanism(s) underlying the complex interaction between DM and CKD; this was done via protein–protein interaction network analysis. Knowledge of how various SNPs and the interaction of metabolites are associated with the CKD and DKD phenotype provides researchers with insights into possible genetic predispositions for these diseases. Moreover, the development of AI-assisted models in this study will advance the ability to carry out precision diagnosis and the molecular classification of DM, CKD, and DKD; this in turn will help the prevention of these diseases and thus will benefit clinical practice in general.

## Methods

### Study participants and sample preparation

Between August 2013 and November 2019, 618 prospectively recruited participants within the Northeastern Taiwan Community Medicine Research Cohort (ClinicalTrials.gov: NCT04839796) were enrolled in this study. Community members who were aged over 30 years old were included in the study after obtaining individual informed consent. Subjects who were pregnant, who were undergoing dialysis therapy, or who had undergone renal transplantation, were excluded. At recruitment, all participants provided a detailed personal history and received a clinical examination. An independent cohort (178 subjects) that was used for external validation was collected from individuals who attended outpatient clinics at Chang Gung Memorial Hospital in 2019 and 2020; the same inclusion and exclusion criteria mentioned above were used for these individuals. Demographic information was collected by questionnaire. Fasting blood and spot urine samples were collected for biochemistry analysis. This study protocol conforms to the ethical guidelines of the 1975 Declaration of Helsinki and was approved by the Institutional Review Board of Chang Gung Medical Foundation (IRB No: 201800802B0, 202000077B0A3, 201800273B0C602, 202002535B0). Written informed consent was obtained from all subjects involved in the study.

### Clinical definitions

Type 2 DM was defined as a fasting glucose of ≥126 mg/dL, a glycosylated hemoglobin ≥6.5, or the use of hypoglycemic medications. Blood pressure was measured using the average of two seated measurements. Hypertension was defined if the patient was receiving medical therapy for such a condition or if their blood pressure was >140/90 mmHg. A body mass index (BMI) of 30 kg/m^2^ or more, which was calculated as weight divided by height^2^, was defined as obesity^43^. CKD was defined using the National Kidney Foundation: Kidney Disease Outcomes Quality Initiative classification with a persistent proteinuria or an eGFR of less than 60 mL/min/1.73 m^2^, as determined by the abbreviated Modification of Diet in Renal Disease equation^44^. Proteinuria was defined if the individual’s urine protein to creatinine ratio was ≥150 mg/g or the individual’s urine albumin to creatinine ratio was ≥ 30 mg/g. DKD was diagnosed if subjects fulfilled both DM criteria and CKD criteria at the same time. Current smoking status was defined as having smoked more than 100 cigarettes in their lifetime and having smoked in the one month before enrollment.

### Biochemical analyses of blood and urine

Peripheral venous blood was obtained after an overnight fast. After centrifugation in 1000 g, the plasma component of the blood was used for either immediate biochemistry analysis or for storage at −80 °C for the subsequent measurements. Genomic DNA was then isolated from peripheral white blood cells using the phenol/chloroform (Sigma- Aldrich, 77607; J.T.Baker, 9180-01) DNA extraction method after lysis of red blood cells. Finally, the DNA from each subject was precipitated and washed using 95% isopropanol (Merck, 1.01040.4000), followed by 80% alcohol (Merck, 1.00983.2500); the resulting DNA was used as total genomic DNA of each individual. Various clinical parameters were determined, including complete blood cell count, liver biochemical marker levels, renal biochemical marker levels, lipid profile, fasting sugar level intact parathyroid hormone level and total 25 (OH) vitamin D level. Serum creatinine was assessed by spectrophotometric analysis using a modified kinetic Jaffe reaction with standardization of the creatinine calibration by an isotope dilution mass spectrometry reference measurement procedure. Electrolyte levels (sodium, potassium, chloride) and carbon dioxide level were assessed using ion-selective electrode methods. Serum calcium and phosphate were measured by spectrophotometric methods (cobas, 05061482190 and 03183793122). Serum albumin and uric acid levels were assessed by colorimetric methods (cobas, 03183688122 and 03183807190). Blood urea nitrogen, was measured conductometry (cobas, 04460715190). Serum intact parathyroid hormone and vitamin D were measured by electrochemiluminescence immunoassay (cobas, 07251068190 and 07464215190). Lipid profiles were obtained by enzymatic methods (cobas, 07005717190, 07528566190, 03039773190, 20767107322). Hemoglobin concentrations were obtained by the cyanide-free sodium lauryl sulphate-Hb spectrophotometric method (sysmex, BJ350971). Urine protein and albumin levels were quantified by colorimetric methods (cobas, 03183734190).

### Untargeted metabolomics and targeted lipidomic analysis

The plasma samples were collected and then they were extracted using methanol before there use in both the untargeted metabolomics analysis and an analysis by commercially available kit (targeted p180 lipidomic analysis, Biocrates, R043-WT20431).

For the untargeted metabolomics analysis using ultra-high performance liquid chromatography-time-of-fly mass spectrometry (UPLC-TOF/MS), in total, 50 μL plasma and 200 μL of cooled methanol were mixed to precipitate any protein present. After centrifugation at 12,000 g for 15 min, the supernatant after transfer was dried using nitrogen gas. The residue was then dissolved in 200 μL 50% acetonitrile for LC-MS analysis. Liquid chromatographic separation was achieved on an ACQUITY UPLC BEH Amide column (1.7 μm, 2.1 × 150 mm, Waters Corp.; Milford, MA, USA) using an ACQUITY TM Ultra Performance Liquid Chromatography (UPLC) system (Waters Corp.). The column was maintained at 45 °C, and the flow rate was set at 0.4 mL/min. The mobile phase A was 0.1% formic acid in water and mobile phase B was acetonitrile containing 0.1% formic acid. Mass spectrometry was performed on a Waters Q Tof-MS (SYNAPT G2S, Waters MS Technologies, Manchester, UK) operated in ESI positive and negative ion modes. The scan range was from 50 to 1000 m/z. The desolvation gas flow was 800 L/hr at 500 °C. The source cone voltage was 25 V. The capillary voltage was 2.5 kV in the positive mode and 2 kV in the negative mode. The lock mass was leucine encephalin (m/z 120.0813 and 556.2771 for positive and m/z 236.1035 and 554.2615 for negative).

For the targeted lipidomic analysis using ultra-high performance liquid chromatography-tandem mass spectrometry (UPLC-MS/MS), the plasma samples were analyzed using a commercially available kit (AbsoluteIDQ p180—BIOCRATES Life Sciences AG, Austria). The targeted 184 metabolites included amino acids, biogenic amines, glycerophospholipids, sphingolipids, acyl carnitines, and hexose and all of these molecules were quantified. The samples were processed as previously described (3). The biogenic amines and amino acids were determined by LC-MS/MS, and the other lipid species were quantified by flow injection analysis coupled with tandem mass spectrometry (FIA-MS/MS). The analysis was performed in positive electrospray ionization mode using a Waters tandem mass spectrometer (TQS, Waters MS Technologies, Manchester, UK). Chromatographic separation was performed on an Acquity BEH C8 column (75 mm × 2.1 mm, particle size of 1.7 μm; Waters crop., Milford, USA) at 50 °C using a linear gradient that ranged from 0.2% formic acid in water to 0.2% formic acid in acetonitrile at a flow rate of 0.9 mL/min. The capillary was set at 3.2 kV. The desolvation gas flow was 1200 L/h at 650 °C. The source temperature was 150 °C and the cone voltage was 10 V. For FIA analysis, 0.03 mL/min was used with a commercial solvent, and the capillary was set at 3.9 kV; the desolvation gas flow was 650 L/h and 350 °C, while the source temperature was 150 °C and the cone voltage was 20 V. All data was processed and analyzed using MetIQ software (Biocrates Life Science AG, Innskruck, Austria). During further analysis, metabolites with >10% missing values, as well as values below the limit of detection (LOD), were excluded. Data on a total of 147 metabolites from five compound classes were collected and these consisted of: 15 acylcarnitines, 21 amino acids, 9 biogenic amines, 88 glycerophospholipids and 14 sphingolipids. All values were processed by median normalization and log2 transformation as appropriate.

### Whole-genome SNP analysis

The genomic DNA was collected from peripheral venous blood and each subject was genotyped using AxiomTM Genome-Wide TWB 2.0 array plates (Thermo Fisher Scientific, 550976). After excluding those with a minor allele frequency rate of 0 or SNPs with a missing rate of more than 10%, a total of 392,885 SNPs were available for further analysis.

### AI-assisted discovery of candidate biomarkers

For the model training and testing, all machine learning analyses were performed with R Version 4.2.1 (using the random Forest, e1071, glmnet, rpart, caret, xgboost and cvAUC packages) and Python 3.7.11 (scikit-learn and xgboost packages). First, we defined three models (Model 1, Model 2 and Model 3) for differentiating DM and CKD using two-stage model building. Model 1 was used to identify DM based on metabolomes, SNPs and clinical tests. Model 2 was used to identify CKD in DM patients based on metabolomes and SNPs. Model 3 was used to identify CKD in non-DM patients based on metabolomes and SNPs. We carried out the same steps for each model using the following feature selection methods.

Before feature selection, a correlation analysis of DM and CKD based on the metabolomes and SNPs was tested by the One-Way Analysis of Variance (ANOVA) or χ2 test (Supplementary Fig. 1). During the feature processing, untargeted metabolites with p-values < 0.05/13231 among the 13231 untargeted metabolites were used for correlation analysis for DM and CKD; the p-values were calculated by ANOVA and adjusted using the Bonferroni correction. In a similar manner, targeted lipidomics information (P180-metabolites) with p-values < 0.05/147 among 147 P180-metabolites, and SNPs with p-values < 0.05/392885 and an odds ratio (OR) >1 among the included 392885 SNPs were used for two separate correlation analyses of DM and CKD (Supplementary Fig. 1a, b). Features were selected by AI-based methods using the following three machine learning algorithms: (1) feature importance using the Random Forest (RF) approach; (2) weighted support vector using the Support Vector Machine (SVM) approach; and^45^ a shrinkage coefficient > 0 using the Least Absolute Shrinkage and Selection Operator (LASSO) approach. Subsequently, the results obtained from these three algorithms were integrated to produce feature importance ranking lists for the three models, namely Model 1, Model 2 and Model 3 (Supplementary Fig. 1c).

Then, we used three supervised algorithms to select important features, namely the RF, SVM and LASSO methods, using an input dataset having a train-to-validation split ratio of 80:20. The metabolomes, SNPs and clinical tests were ranked based on the summation of the selected counts using 100-time bootstrapped random samples and the three machine-learning methods. The three models (Model 1, Model 2 and Model 3) were used to extract the minimum features required for highest performance in terms of AUC and accuracy rate (Supplementary Figs. 2, 4). After this process, we retained the known features with defined identity based on their feature importance from the three models (Model 1, Model 2 and Model 3). Finally, we used 10-fold cross validations (random sample with a train and test split ratio of 90:10) to carry out two-stage model building using Extremely Randomized Trees (Extra-Tree), RF, SVM, Logistic Regression (LR) and Extreme Gradient Boosting (XGB). Furthermore, the validation cohort was used to valid our training model to avoid overfitting (Fig. 1e and Supplementary Table 8).

### Statistical methods

The ANOVA was used to compare differences between the continuous variables that were derived from more than two groups. The results are presented as medians (Min, Max) or means (standard deviations). The χ^2^ test was used to examine the distribution of categorical variables. The *p*-values of the ANOVA and χ^2^ tests were used to determine the strength of the association of variables with control status, DM, non-diabetic CKD and DKD; we also investigated the distribution of these groups. Univariate logistic regression analysis followed by backward selection multivariate logistic regression analysis was conducted to determine the associations between a given AI-discovered feature and the various different disease groups. The statistical software used for this study was R (version 4.2.1).

### Reporting summary

Further information on research design is available in the Nature Research Reporting Summary linked to this article.

## Supplementary information

SUPPLEMENTAL MATERIAL

Reporting Summary

## Data Availability

The SNP datasets generated during and/or analysed during the current study are available in the Gene Expression Omnibu (GEO) repository, **accessi**on code: GSE215221. The metabolomics data supporting the findings of this study are available from the corresponding author upon reasonable request.

## Code availability

The code in this study is available from the authors upon reasonable request.
