# Supplementary material for: Discovering a trans-omics biomarker signature that predisposes high risk diabetic patients to diabetic kidney disease
Source: NPJ Digit Med. 2022 Nov 2;5:166. doi: 10.1038/s41746-022-00713-7 (PMC9630270; doi:10.1038/s41746-022-00713-7)
Supplement: Supplementary file 1 — SUPPLEMENTAL MATERIAL [file 41746_2022_713_MOESM1_ESM.pdf]

Supplementary Information

Discovery of a Trans-Omics Biomarker Signature that Predisposes High Risk Diabetic Patients to Diabetic Kidney Disease

I-Wen Wu[1,2,3], Tsung-Hsien Tsai[4], Chi-Jen Lo[5], Yi-Ju Chou[6], Chi-Hsiao Yeh[2,3,7], Yun-Hsuan Chan[4], Jun-Hong Chen[4], Paul Wei-Che Hsu[6], Heng-Chih Pan[1,2], Heng-Jung Hsu[1,2], Chun-Yu Chen[1,2], Chin-Chan Lee[1,2], Yu-Chiau Shyu[2,8], Chih-Lang Lin[2,9], Mei-Ling Cheng[5,10,11], Chi-Chun Lai[2,3,12]*, Huey-Kang Sytwu[13,14]*, Ting-Fen Tsai[6,15,16]*

[1]Department of Nephrology, Chang Gung Memorial Hospital, Keelung 204, Taiwan;
[2]Community Medicine Research Center, Chang Gung Memorial Hospital, Keelung 204, Taiwan;
[3]College of Medicine, Chang Gung University, Taoyuan 333, Taiwan;
[4]Advanced Tech BU, Acer Inc., New Taipei City 221, Taiwan;
[5]Metabolomics Core Laboratory, Healthy Aging Research Center, Chang Gung University, Taoyuan City 333, Taiwan;
[6]Institute of Molecular and Genomic Medicine, National Health Research Institutes, Zhunan 350, Taiwan;
[7]Department of Thoracic and Cardiovascular Surgery, Chang Gung Memorial Hospital, Linkou 333, Taiwan;
[8]Department of Nursing, Chang Gung University of Science and Technology, Taoyuan 333, Taiwan;
[9]Department of Gastroenterology and Hepatology, Chang Gung Memorial Hospital, Keelung 204, Taiwan;
[10]Clinical Metabolomics Core Laboratory, Chang Gung Memorial Hospital, Taoyuan City 33302, Taiwan.
[11]Department of Biomedical Sciences, College of Medicine, Chang Gung University, Taoyuan City 33302, Taiwan.
[12]Department of Ophthalmology, Chang Gung Memorial Hospital, Keelung 204, Taiwan
[13]National Institute of Infectious Diseases and Vaccinology, National Health Research Institutes, Zhunan 350, Taiwan
[14]Department & Graduate Institute of Microbiology and Immunology, National Defense Medical Center, Taipei 114, Taiwan;
[15]Department of Life Sciences and Institute of Genome Sciences, National Yang Ming Chiao Tung University, Taipei 112, Taiwan;
[16]Center for Healthy Longevity and Aging Sciences, National Yang Ming Chiao Tung University, Taipei 112, Taiwan

Correspondence: chichun.lai@gmail.com (C.-C.L.); sytwu@nhri.edu.tw (H.-K.S.); tftsai@ym.edu.tw (T.-F.T.); Tel.: +886-2-24313131 ext. 6101 (C.-C.L.); +886-37-206166 ext. 31010 (H.-K.S.); +886-2-28267293 (T.-F.T.)

Supplementary Tables

Supplementary Tables 1. Baseline characteristics of training cohort and testing cohort

Parameters	All (n = 796)	Training cohort (n = 557)	Testing cohort (n = 61)	Validation cohort (n=178)
Age, years	63.1 ± 13.2	64.0 ± 12.7	62.2 ± 14.3	60.6 ± 14.2
Male, No. (%)	367 (46.1%)	259 (46.5%)	28 (45.9%)	80 (44.9%)
**Comorbidities**				
Diabetes, No. (%)	263 (33.0%)	187 (33.6%)	20 (32.8%)	56 (31.5%)
Hypertension, No. (%)	328 (41.2%)	247 (44.3%)	21 (34.4%)	60 (33.7%)
Obesity, No. (%)	537 (67.5%)	406 (72.9%)	35 (57.4%)	96 (53.9%)
**Personal habits**				
Smoking, No. (%)	190 (23.9%)	147 (26.4%)	20 (32.8%)	23 (12.9%)
Alcohol drinking, No. (%)	250 (31.4%)	190 (34.1%)	26 (42.6%)	34 (19.1%)
**Anthropometrics**				
Body mass index, kg/m2	26.3 ± 4.3	26.6 ± 4.0	26.9 ± 4.9	25.2 ± 4.6
Systolic BP, mmHg	134.1 ± 45.1	133.8 ± 17.4	132.7 ± 17.4	135.6 ± 90.1
Diastolic BP, mmHg	77.2 ± 11.0	77.8 ± 11.1	77.6 ± 10.4	75.4 ± 10.9
**Laboratory**				
eGFR, mL/min per 1.73 m2 (MDRD)	83.4 (0.4, 171.7)	82.8 (8.6, 171.7)	86.0 (11.9, 145.9)	84.1 (0.4, 157.3)
BUN, mg/dL	15.4 (0.7, 129.0)	15.6 (6.3, 54.6)	15.0 (5.8, 75.4)	14.0 (0.7, 129.0)
Serum creatinine, mg/dL	0.8 (0.4, 100.6)	0.8 (0.4, 6.9)	0.8 (0.5, 4.7)	0.8 (0.4, 100.6)
Serum albumin, g/dL	4.6 (0.7, 246.4)	4.6 (3.1, 246.4)	4.6 (3.6, 5.3)	4.5 (0.7, 5.2)
Cholesterol, mg/dL	188 (4, 377)	187 (92, 377)	191 (99, 279)	189 (4, 313)
Triglycerides, mg/dL	116 (21, 1225)	121 (25, 1225)	119 (35, 409)	99 (21, 530)
hs-CRP, mg/L	1.1 (0.1, 73.1)	1.1 (0.2, 73.1)	1.0 (0.1, 59.6)	1.3 (0.2, 66.0)
Urine albumin/creatinine ratio, mg/g	9.9 (0.9, 5708.6)	10.4 (1.3, 2085.0)	7.9 (2.1, 3792.1)	6.9 (0.9, 5708.6)
Vitamin D, ug/mL	580.1 (22.3, 3499.0)	572.5 (22.3, 3442.0)	530.9 (114.0, 3374.0)	609.4 (159.6, 3499.0)
iPTH, pmol/L	42.0 (6.0, 199.0)	42.3 (6.0, 199.0)	40.4 (17.4, 99.9)	42.0 (13.7, 195.0)
Serum calcium, mg/dL	9.4 (6.6, 10.5)	9.4 (6.6, 10.5)	9.4 (8.1, 10.2)	9.4 (8.1, 10.4)
Serum phosphate, mg/dL	3.6 (2.1, 5.7)	3.6 (2.1, 5.7)	3.6 (2.8, 4.8)	3.6 (2.3, 5.2)
Insulin, uU/mL	10.4 (0.5, 176.0)	11.0 (1.2, 84.9)	10.3 (0.5, 43.5)	8.2 (1.5, 176.0)
LDL-C / HDL-C, mg/dL	2.2 (0.6, 5.9)	2.3 (0.6, 5.9)	2.5 (0.8, 4.6)	2.1 (0.6, 4.5)
UreaNU, mg/dL	794.2 (1.6, 1923.3)	829.2 (117.6, 1923.3)	751.7 (152.7, 1546.7)	712.9 (1.6, 1652.0)
Glycated Hemoglobin, %	5.9 (4.5, 106.0)	6.0 (4.5, 14.4)	5.9 (4.8, 11.1)	5.8 (4.7, 106.0)
Glucose, mg/dL	100.0 (67.0, 400.0)	102.0 (69.0, 400.0)	99.0 (82.0, 218.0)	96.0 (67.0, 393.0)
**Grouping**				
Normal control, No. (%)	438 (55.0%)	302 (54.2%)	36 (59.0%)	100 (56.2%)
Diabetes, No. (%)	132 (16.6%)	96 (17.2%)	10 (16.4%)	26 (14.6%)
Non-diabetic CKD, No. (%)	95 (11.9%)	68 (12.2%)	5 (8.2%)	22 (12.4%)
Diabetic kidney disease, No. (%)	131 (16.5%)	91 (16.3%)	10 (16.4%)	30 (16.9%)

The values are expressed as means ± SD or median (Min, Max) or n (%). Abbreviations: CKD, chronic kidney disease; BUN, blood urea nitrogen; eGFR, estimated glomerular filtration rate; hs-C reactive protein, high-sensitivity C reactive protein; LDL-C / HDL-C, low density lipoprotein-cholesterol / high density lipoprotein-cholesterol.

Supplemental Table 2. Feature Importance for predicting DM (model 1)

Rank	Feature	Category	Comment
1	Acetylcarnitine	Metabolite	carnitine
2	Fucose	Metabolite	hexose
3	Mannose/Inositol I	Metabolite	Aldohexose
4	Age	Clinical	
5	Sulfanilamide	Metabolite	drug
6	Proline	Lipidomic	Amino acid
7	Insulin	Clinical	
8	LDL-C-direct-	Clinical	
9	PC_{ae} 34:2	Lipidomic	plasmalogen
10	lysoPC 18:0	Lipidomic	lyso-phospholipid
11	Serine	Lipidomic	Amino acid
12	Sarcosine	Lipidomic	Biogenic amine
13	Valerylcarnitine	Lipidomic	carnitine
14	Alanine	Lipidomic	Amino acid
15	PC_{ae} 38:6	Lipidomic	plasmalogen
16	Aspartate	Lipidomic	Amino acid
17	rs3755899	SNP	Non Coding Transcript Variant: UGDH-AS1
18	Propionylcarnitine	Lipidomic	carnitine
19	rs933229	SNP	Intron Variant: LOC105373021
20	PC_{ae} 32:2	Lipidomic	plasmalogen

Supplemental Table 3. Feature Importance for predicting CKD in DM patients (model 2)

Rank	Feature	Category	Comment
1	Serine	Lipidomic	Amino acid
2	Resolvin D1	Metabolite	
3	Pseudouridine	Metabolite	
4	Kynurenine	Lipidomic	Biogenic amine
5	Arabitol	Metabolite	
6	PC ae C30:0	Lipidomic	
7	Symmetric dimethylarginine	Lipidomic	Biogenic amine
8	rs1868138	SNP	ALDH1L1
9	rs184518892	SNP	LY6D
10	rs117681509	SNP	PCDH9

Supplemental Table 4. Feature Importance for predicting CKD in non-DM patients (model 3)

Rank	Feature	Category	Comment
1	Mannose/Inositol II	Metabolite	hexose
2	Symmetric dimethylarginine	Lipidomic	plasmalogen
3	Kynurenine	Lipidomic	Biogenic amine
4	Mannose/Inositol I	Metabolite	hexose
5	Uridine	Metabolite	pyrimidine nucleoside
6	Alanine	Metabolite	Amino acid
7	Citrulline	Lipidomic	Amino acid
8	Acetyl neuraminic acid	Metabolite	Neuraminic acid
9	Cystine	Metabolite	Amino acid
10	rs898097	SNP	Intron Variant: B3GNTL1
11	Asymmetric dimethylarginine	Lipidomic	Biogenic amine
12	Age	Clinical	
13	rs11097023	SNP	Intron Variant: CDS1
14	Acetylcarnitine	Lipidomic	carnitine
15	rs2856966	SNP	Missense Variant: ADCYAP1
16	Mannitol	Metabolite	drug
17	Serine	Lipidomic	Amino acid
18	rs28533579	SNP	Intron Variant: FAM53A
19	PC_{ae} 30:2	Lipidomic	plasmalogen
20	Butyrylcarnitine	Lipidomic	carnitine
21	rs12451753	SNP	Synonymous Variant: CCDC182
22	Cysteic acid	Metabolite	taurine metabolism
23	PC_{ae} 36:2	Lipidomic	plasmalogen
24	PC_{aa} 36:0	Lipidomic	phosphatidylcholine
25	BMI	Clinical	

Supplementary Table 5. Cross-validation of prediction performance in Model 1

	Model	Target	Sensitivity	Specificity	PPV	NPV	Accuracy	AUC	Cut Point
**Model1**	**Extremely Randomized Trees**	**DM**	**0.72**	**0.88**	**0.75**	**0.86**	**0.83**	**0.89**	0.5
	Logistic Regression		0.76	0.83	0.69	0.87	0.80	0.87	
	Random Forest		0.73	0.86	0.73	0.87	0.82	0.88	
	Extreme Gradient Boosting		0.74	0.84	0.70	0.86	0.81	0.88	
	Support Vector Machine		0.75	0.83	0.69	0.87	0.81	0.88	

Supplementary Table 6. Cross-validation of prediction performance in Model 2

	Model	Target	Sensitivity	Specificity	PPV	NPV	Accuracy	AUC	Cut Point
**Model2**	Extremely Randomized Trees	**CKD**	0.64	0.72	0.70	0.67	0.68	0.73	0.5
	Logistic Regression		0.63	0.81	0.77	0.69	0.72	0.74	
	Random Forest		0.69	0.64	0.66	0.67	0.67	0.71	
	Extreme Gradient Boosting		0.64	0.69	0.67	0.66	0.67	0.72	
	Support Vector Machine		0.67	0.73	0.71	0.69	0.70	0.77	
	**Ensemble (Mean)**		**0.67**	**0.73**	**0.71**	**0.69**	**0.70**	**0.76**	

Supplementary Table 7. Cross-validation of prediction performance in Model 3

	Model	Target	Sensitivity	Specificity	PPV	NPV	Accuracy	AUC	Cut Point
**Model3**	**Extremely Randomized Trees**	CKD	**0.47**	**0.90**	**0.50**	**0.89**	**0.82**	**0.76**	0.5
	Logistic Regression		0.59	0.79	0.38	0.9	0.75	0.76	
	Random Forest		0.38	0.91	0.48	0.87	0.82	0.77	
	Extreme Gradient Boosting		0.42	0.88	0.42	0.88	0.79	0.76	
	Support Vector Machine		0.55	0.79	0.37	0.89	0.75	0.75	

Supplementary Table 8. Performance of the three models on the validation cohort

Model 1	Model	Target	Sensitivity	Specificity	PPV	NPV	Accuracy	AUC	Cut Point
**Training cohort (internal 10-folds validation)**	Extremely Randomized Trees	DM	0.72	0.88	0.75	0.86	0.83	0.89	0.5
**External validation cohort**	Extremely Randomized Trees		0.71	0.89	0.75	0.87	0.84	0.91	

Model 2	Model	Target	Sensitivity	Specificity	PPV	NPV	Accuracy	AUC	Cut Point
**Training cohort (internal 10-folds validation)**	Ensemble (Mean of five models)	CKD	0.67	0.73	0.71	0.69	0.70	0.76	0.5
**External validation cohort**	Ensemble (Mean of five models)		0.67	0.70	0.74	0.62	0.68	0.71	

Model 3	Model	Target	Sensitivity	Specificity	PPV	NPV	Accuracy	AUC	Cut Point
**Training cohort (internal 10-folds validation)**	Extremely Randomized Trees	CKD	0.47	0.90	0.50	0.89	0.82	0.76	0.5
**External validation cohort**	Extremely Randomized Trees		0.32	0.93	0.50	0.86	0.82	0.78	

Supplementary Table 9. Expression levels of mRNA of candidate genes

Model	Gene Symbol	Gene Name	Expression Level of mRNA* (NX)					
			kidney	pancreas	liver	adipose	heart	others
**Model 1 Feature associated with DM**	**RPTOR**	Regulatory-Associated Protein Of MTOR	14.3	9.8	12.9	13.6	15.5	ubiquitous
	**CLPTM1L**	Cleft Lip And Palate Transmembrane Protein 1-Like Protein	29.4	57.6	56.1	15.8	13.1	ubiquitous
**Model 2 Features associated with CKD in DM patients**	**ALDH1L1**	Aldehyde Dehydrogenase 1 Family Member L1	48.1	10.2	148.7	28.9	8.1	salivary gland (34.1)
	**LY6D**	Lymphocyte Antigen 6 Family Member D	0.2	0.2	0.2	0.2	0.2	tongue (316.2), esophagus (236.9)
	**PCDH9**	Protocadherin 9	3.5	2.0	2.3	4.5	5.7	cerebral cortex (47.0)
**Model 3 Features associated with CKD in non-DM patients**	**B3GNTL1**	UDP-GlcNAc:BetaGal Beta-1,3-N-Acetylglucosaminyltransferase Like 1	2.9	7.6	3.4	3.7	6.2	Ubiquitous
	**CDS1**	CDP-Diacylglycerol Synthase 1	11.9	4.5	0.6	0.9	0.5	Small intestine (48.0)
	**ADCYAP1**	Adenylate Cyclase Activating Polypeptide 1	0.6	7.7	0.3	2.1	1.0	pons and medulla (33.8), appendix (16.5)
	**FAM53A**	Family With Sequence Similarity 53 Member A	0.7	4.0	1.2	1.3	0.5	testis (12.7)

*NX is a normalized expression value from consensus data by databases HPA, GTEx and FANTOM5. After obtaining consensus expression by calculating TPM (transcripts per million), the value is obtained after processing through TMM normalized and Pareto scaling. Data source: The Human Protein Atlas https://www.proteinatlas.org.

Supplementary Table 10. Expression levels of protein of candidate genes.

Model	Gene Symbol	Gene Name	Expression Level of Protein*					
			kidney	pancreas	liver	adipose	heart	others
**Model 1 Feature associated with DM**	**RPTOR**	Regulatory-Associated Protein Of MTOR	High	Medium	Low	Medium	Medium	Ubiquitous
	**CLPTM1L**	Cleft Lip And Palate Transmembrane Protein 1-Like Protein	Medium	High	Medium	Not detected	Not detected	Stomach, Testis
**Model 2 Features associated with CKD in DM patients**	**ALDH1L1**	Aldehyde Dehydrogenase 1 Family Member L1	High	Low	High	Medium	Low	Cerebellum, Testis
	**LY6D**	Lymphocyte Antigen 6 Family Member D	Not detected	Not detected	Not detected	Not detected	Not detected	Esophagus, Skin
	**PCDH9**	Protocadherin 9	-	-	-	-	-	-
**Model 3 Features associated with CKD in non-DM patients**	**B3GNTL1**	UDP-GlcNAc:BetaGal Beta-1,3-N-Acetylglucosaminyltransferase Like 1	Medium	Medium	Not detected	Low	Medium	Testis
	**CDS1**	CDP-Diacylglycerol Synthase 1	Low	Low	Not detected	Low	Medium	Cerebellum
	**ADCYAP1**	Adenylate Cyclase Activating Polypeptide 1	Not detected	Not detected	Not detected	Not detected	Not detected	Pituitary gland
	**FAM53A**	Family With Sequence Similarity 53 Member A	Medium	Low	Not detected	Low	Low	Testis

Supplementary Figures

Supplementary Figure 1

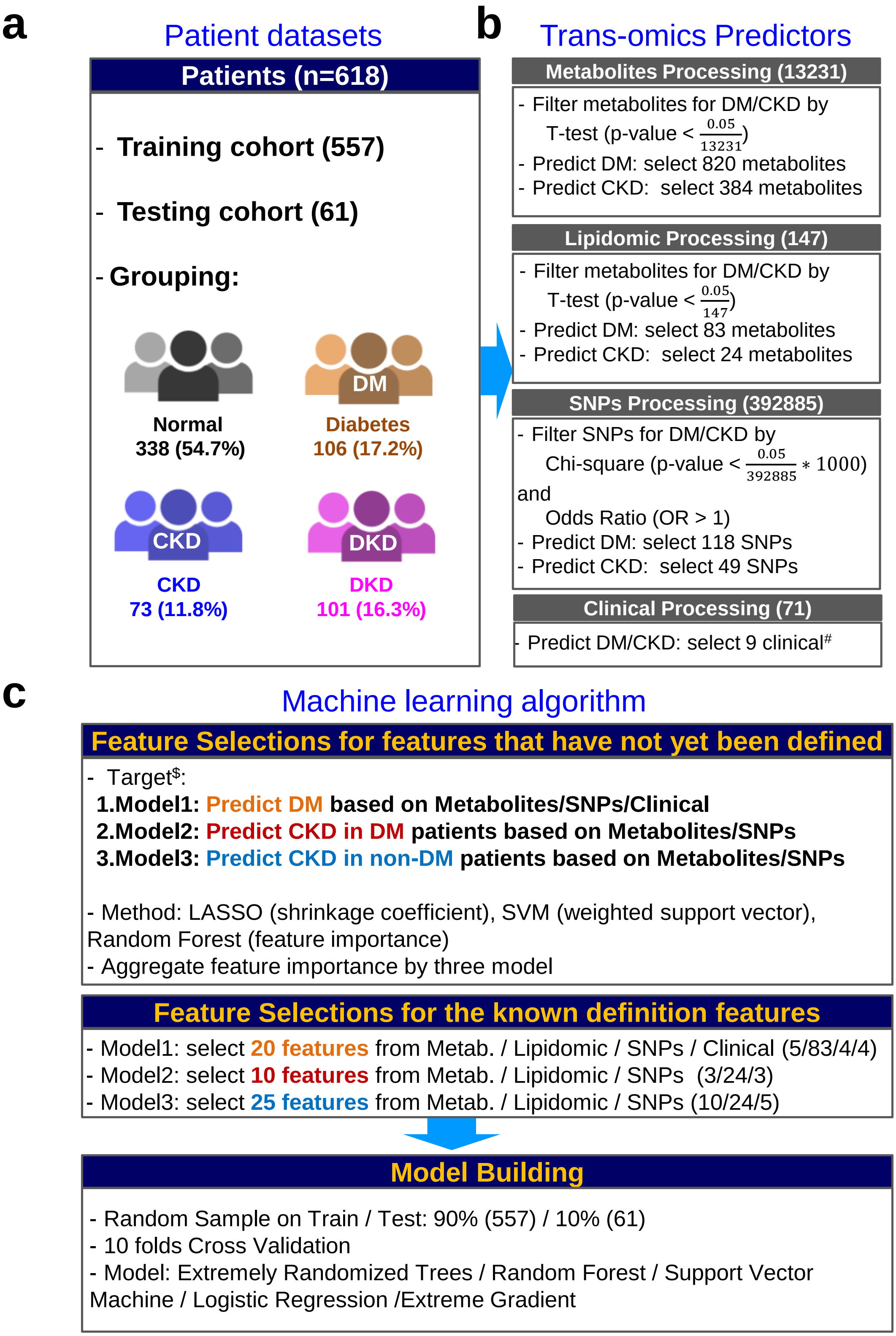

Supplementary Figure 1. Study flow chart, machine learning algorithms and their performances in the three predicting models. (a) (b) Data processing workflows for the integrated analyses of un-target metabolites, lipidomics (P180-metabolites), SNPs, and clinical data. The metabolites contains 13231 un-target metabolites and the Lipidomic recruits 147 known metabolites from the P180 kits. # indicates the 9 clinical features selected by AI, namely Age, BMI, Gender, BUN, Insulin, Intact_PTH, LDL_C_direct, T_Cholesterol, and UreaNU. **(c)** Machine learning algorithm for the selections of best features and model building for the three models. $ indicates that Age and BMI must be the input features of Model 1, 2, 3 for feature selections.

Supplementary Figure 2

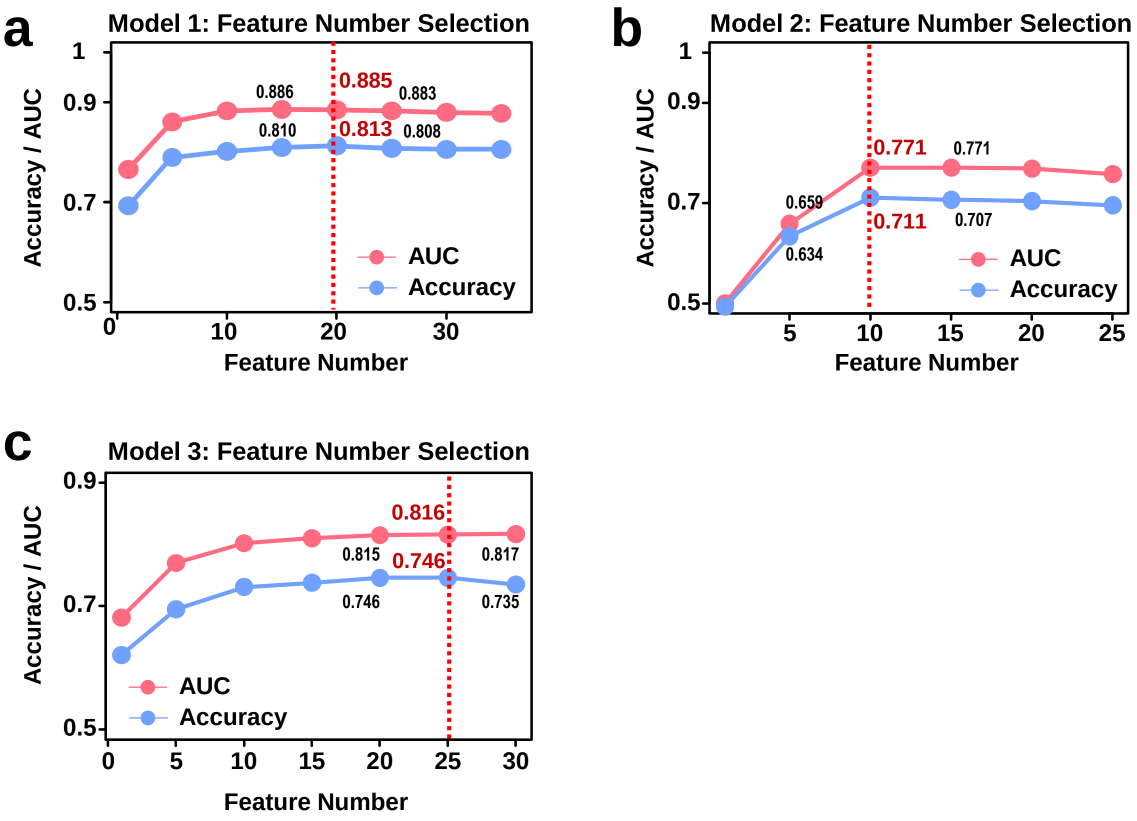

Supplementary Figure 2. The number of feature selection was determined by AUC and accuracy. **(a)** Model 1 (20 features), **(b)** Model 2 (10 features), and **(c)** Model 3 (25 features).

Supplementary Figure 3

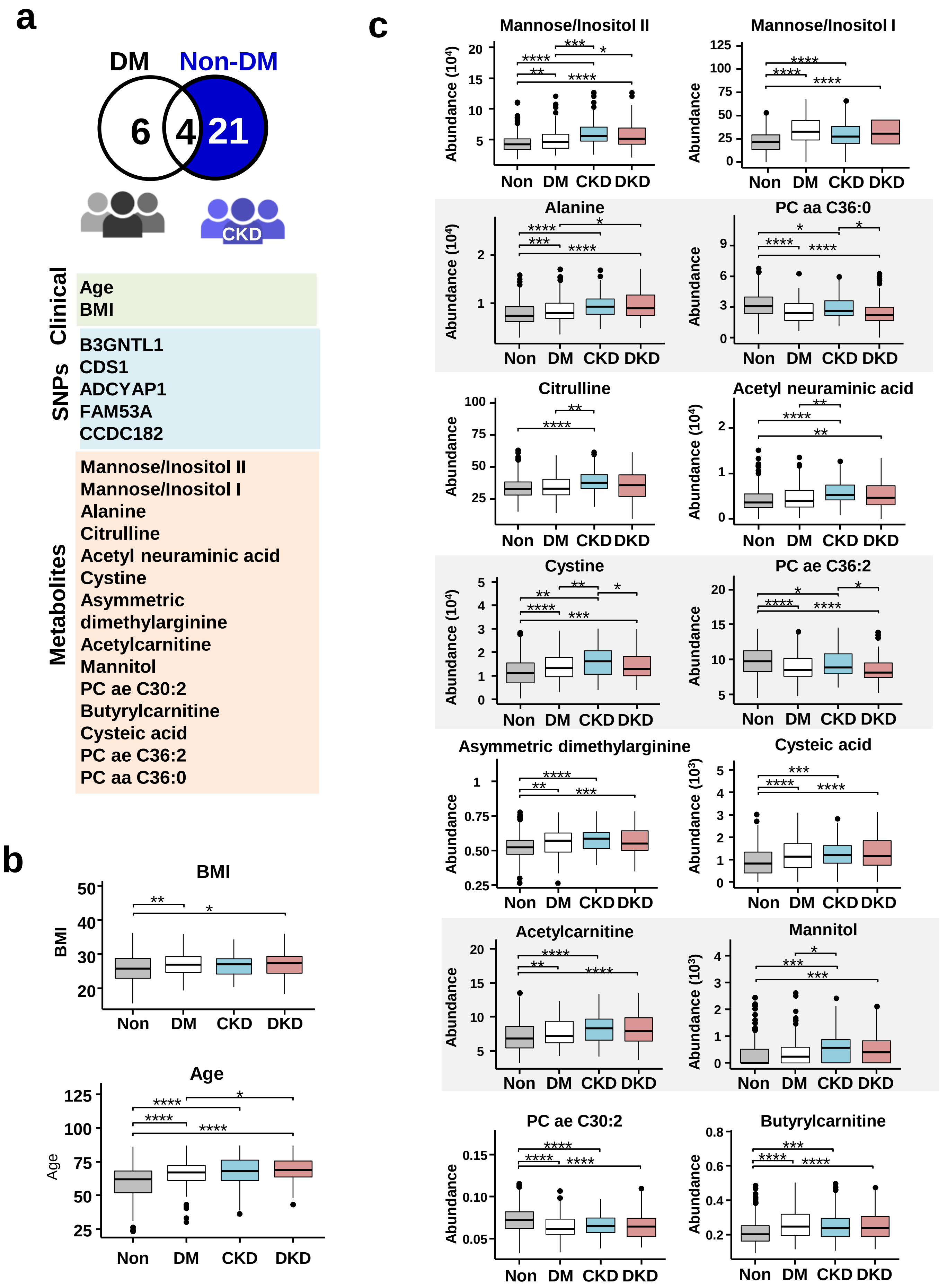

Supplementary Figure 3 (continued)

d

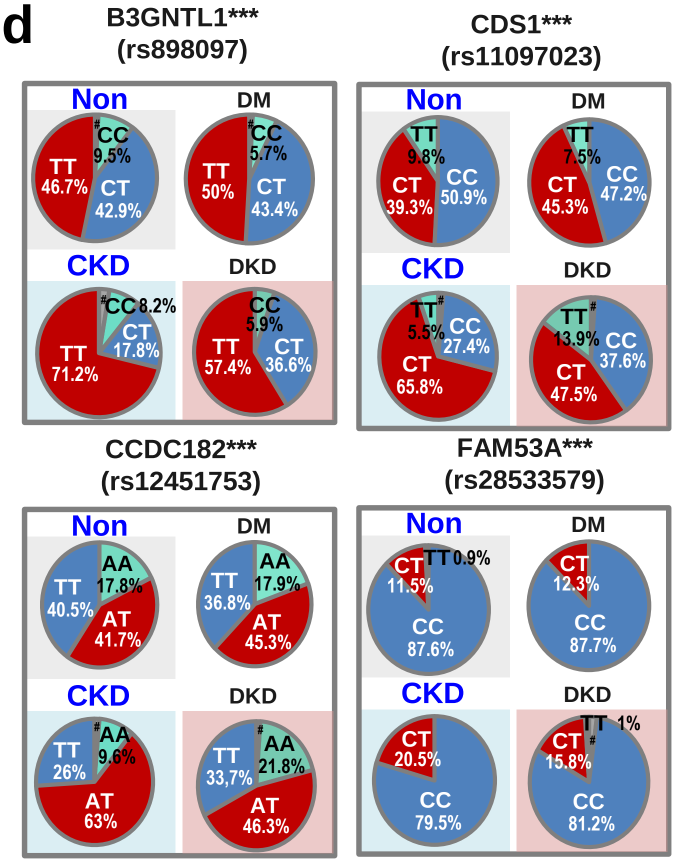

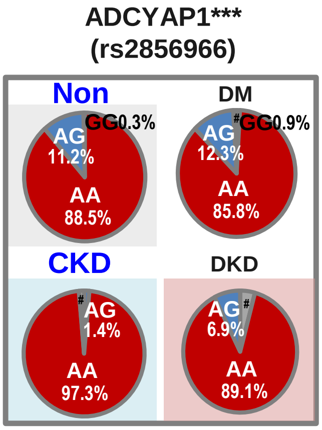

e

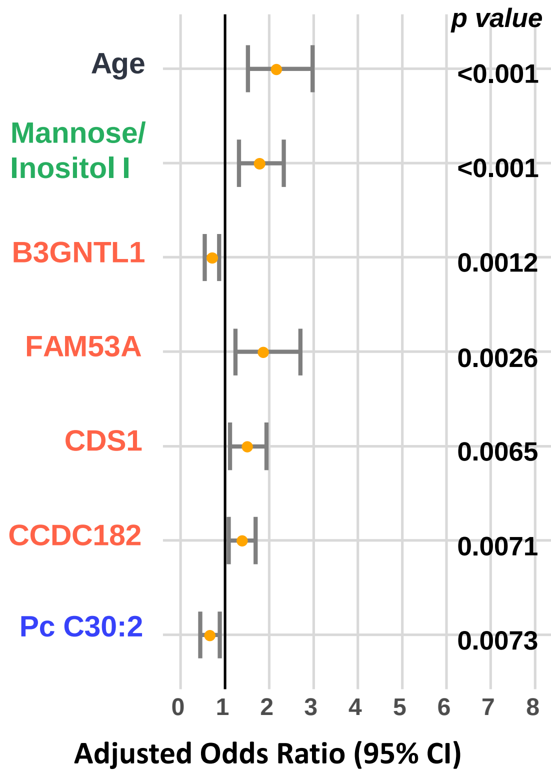

Supplementary Figure 3. Selected features for predicting renal dysfunction in non-DM patients (Model 3). (a) Venn diagram of AI-selected features in model 3. **(b) (c)** Boxplot of the clinical features**(b)** and the metabolite features **(c)** in Model 3. The statistical analysis with *p*-values was performed by ANOVA for significant metabolites in the four groups. The t-test was used for multiple comparisons within the four groups test**.** Box plot: Box plot includes a box and a set of whiskers. The lower line of the box is represented as Q1(25th percentile). The upper line of the box is represented as Q3(75th percentile). The middle and bold line in the box is represented as median. In general, the boundary of the lower and upper whiskers is 1.5 interquartile ranges (IQR, IQR = Q3 - Q1) below the Q1 and 1.5 IQR above the Q3. The extreme values outside this boundary are considered as outliers and plotted as black dots. If all data points are between Q1 - 1.5 x IQR and Q3 +1.5 x IQR, the boundary of the lower and upper whiskers should be minimum and maximum of the data. The error bar here means the lower and upper whiskers that we define above. **(d)** Pie charts indicating the genotype frequencies of SNPs using SNP datasets obtained from the subjects. # indicted the signaling of SNP array was lower than the calling rates. The χ2 test was used for comparisons of genotype frequencies within the four groups. **(e)** Adjusted odds ratios of factors in backward logistic regression procedure associated with the occurrence of CKD in non-DM patients. The Wald test was used to construct 95% confidence interval (CI) and test the significance of adjusted odds ratios of risk factors. The error bar here means the lower bound and upper bound of adjusted odds ratio of 95% confidence interval. **p <0.05, **p <0.01, ***p < 0.001, ****p < 0.0001*. Abbreviations: B3GNTL1, UDP-GlcNAc:BetaGalBeta-1,3-N-Acetylglucosaminyltransferase like 1; CDS1, CDP-Diacylglycerol synthase 1; CCDC182, coiled-coil domain containing 182; FAM53A, family with sequence similarity 53 member A; ADCYAP1, adenylate cyclase activating polypeptide 1.

Supplementary Figure 4

a

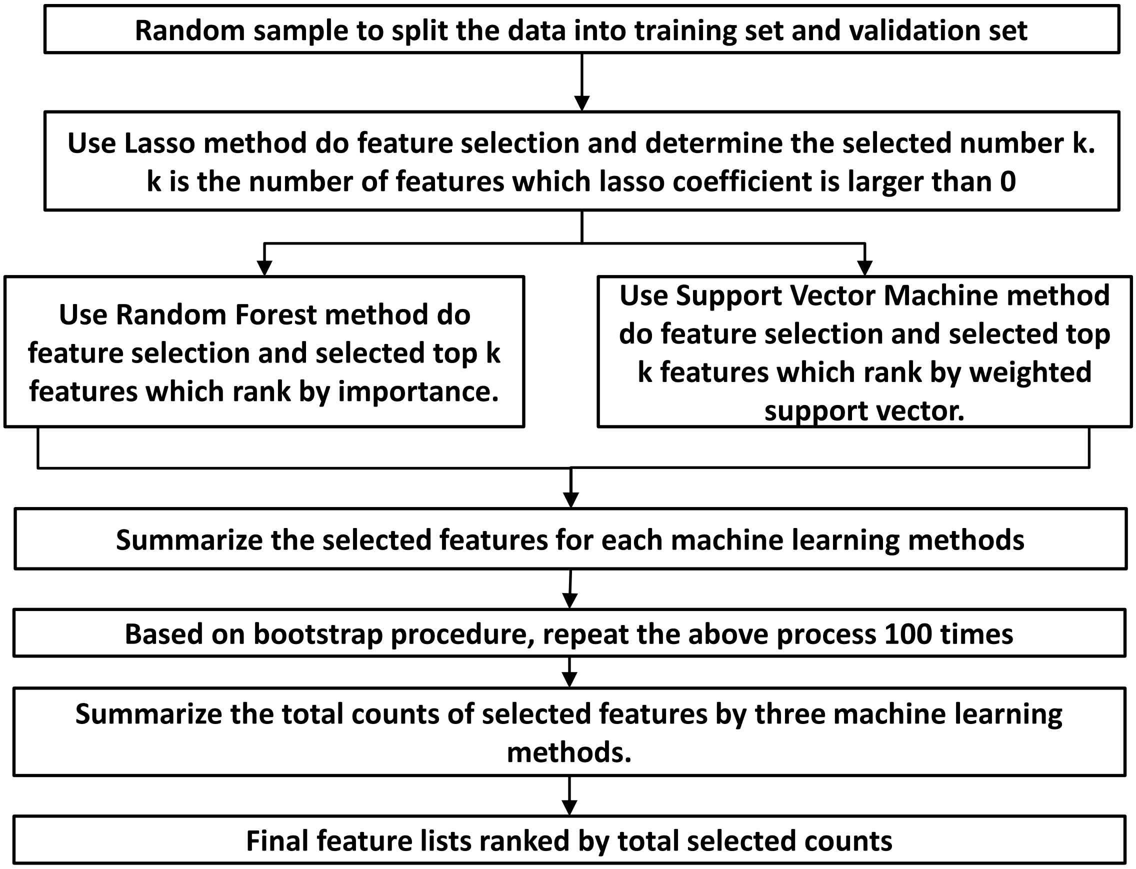

b

Bootstrap the process 100 times

Random sample to split the data into training set and validation set

LASSO feature selection

Rank	Feature	Bootstrap
1	X1	1
2	X2	1
3	X3	1
⋮	⋮	⋮
K	X101	1

SVM feature selection

Rank	Feature	Bootstrap
1	X2	1
2	X3	1
3	X4	1
⋮	⋮	⋮
k	X87	1

RF feature selection

Rank	Feature	Bootstrap
1	X2	1
2	X3	1
3	X5	1
⋮	⋮	⋮
k	X65	1

Rank	Feature	Selection count
1	X2	298
2	X3	295
3	X1	288
4	X15	283
⋮	⋮	⋮

Aggregate feature selection By ranking selection count

Max of selection count = 100(times)*3(method)=300

Supplementary Figure 4 (continued)

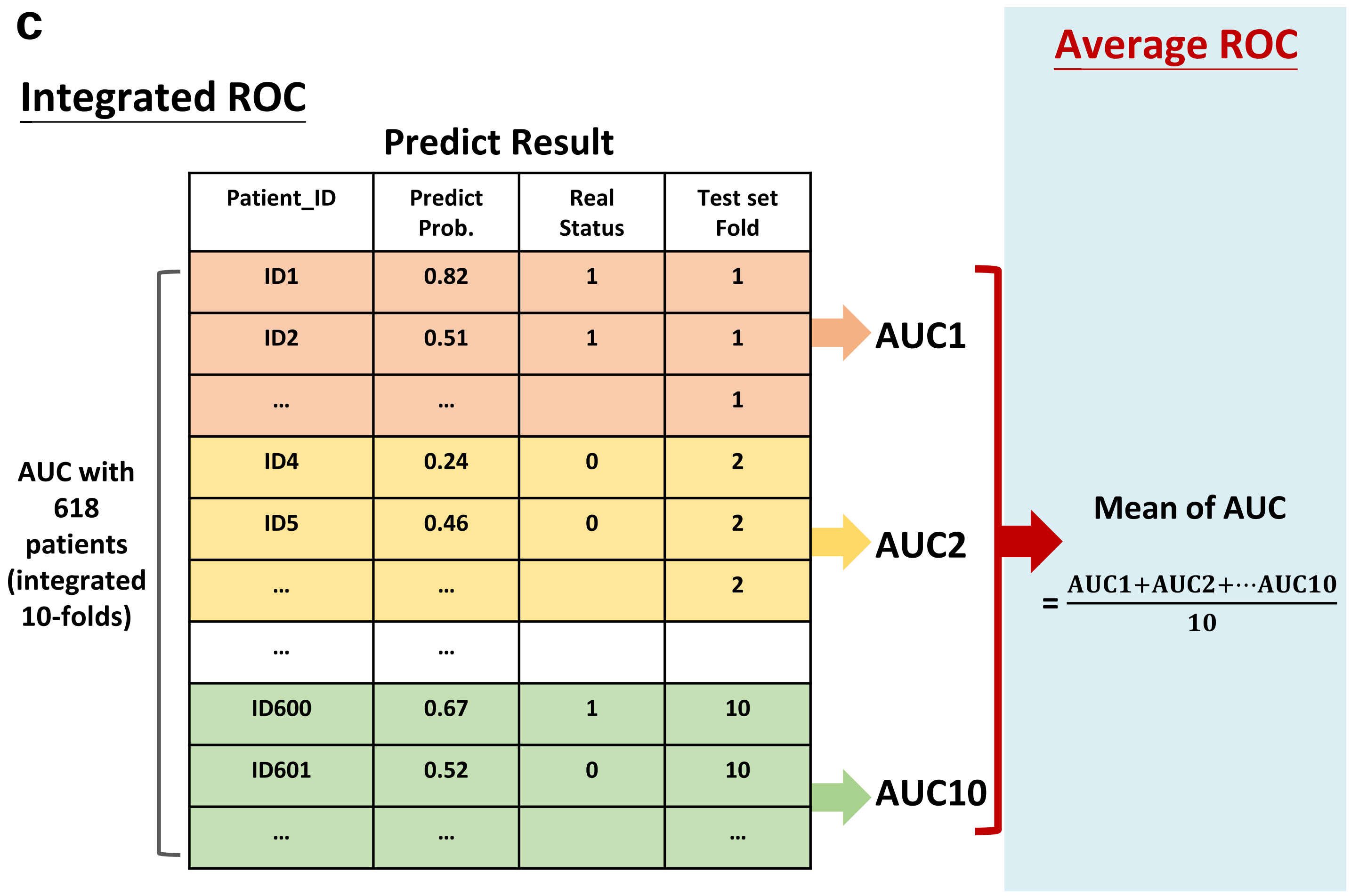

Supplementary Figure 4. (a) The flow chart of integrating three algorithms' results in ranking features. **(b)** Schematic diagram of how to conduct the feature ranking list. **(c)** Schematic diagram of the difference between integrated ROC and average ROC.
