# Supplementary material for: Discovering a trans-omics biomarker signature that predisposes high risk diabetic patients to diabetic kidney disease
Source: NPJ Digit Med. 2022 Nov 2;5:166. doi: 10.1038/s41746-022-00713-7 (PMC9630270; doi:10.1038/s41746-022-00713-7)
Supplement: Supplementary file 2 — Reporting Summary [file 41746_2022_713_MOESM2_ESM.pdf]

nature portfolio

Corresponding author(s): Chi-Chun Lai, Huey-Kang Sytwu, Ting-Fen Tsai

Last updated by author(s): 10/06/2022

Reporting Summary

Nature Portfolio wishes to improve the reproducibility of the work that we publish. This form provides structure for consistency and transparency in reporting. For further information on Nature Portfolio policies, see our Editorial Policies and the Editorial Policy Checklist.

Statistics

For all statistical analyses, confirm that the following items are present in the figure legend, table legend, main text, or Methods section.

n/a	Confirmed	
☐	☒	The exact sample size (*n*) for each experimental group/condition, given as a discrete number and unit of measurement
☐	☒	A statement on whether measurements were taken from distinct samples or whether the same sample was measured repeatedly
☐	☒	The statistical test(s) used AND whether they are one- or two-sided<br>*Only common tests should be described solely by name; describe more complex techniques in the Methods section.*
☐	☒	A description of all covariates tested
☐	☒	A description of any assumptions or corrections, such as tests of normality and adjustment for multiple comparisons
☐	☒	A full description of the statistical parameters including central tendency (e.g. means) or other basic estimates (e.g. regression coefficient) AND variation (e.g. standard deviation) or associated estimates of uncertainty (e.g. confidence intervals)
☐	☒	For null hypothesis testing, the test statistic (e.g. *F*, *t*, *r*) with confidence intervals, effect sizes, degrees of freedom and *P* value noted<br>*Give P values as exact values whenever suitable.*
☒	☐	For Bayesian analysis, information on the choice of priors and Markov chain Monte Carlo settings
☒	☐	For hierarchical and complex designs, identification of the appropriate level for tests and full reporting of outcomes
☒	☐	Estimates of effect sizes (e.g. Cohen's *d*, Pearson's *r*), indicating how they were calculated

Our web collection on statistics for biologists contains articles on many of the points above.

Software and code

Policy information about availability of computer code

Data collection	Not appropriate since our data are collected from blood test or lab data.
Data analysis	The statistical software used for this study was R Version 3.5.3.

For manuscripts utilizing custom algorithms or software that are central to the research but not yet described in published literature, software must be made available to editors and reviewers. We strongly encourage code deposition in a community repository (e.g. GitHub). See the Nature Portfolio guidelines for submitting code & software for further information.

Data

Policy information about availability of data

All manuscripts must include a data availability statement. This statement should provide the following information, where applicable:

- Accession codes, unique identifiers, or web links for publicly available datasets
- A description of any restrictions on data availability
- For clinical datasets or third party data, please ensure that the statement adheres to our policy

Data Availability Statement" as following: The datasets generated during and/or analysed during the current study are available in the Gene Expression Omnibu (GEO) repository, https://www.ncbi.nlm.nih.gov/geo/query/acc.cgi?acc=GSE215221

Field-specific reporting

Please select the one below that is the best fit for your research. If you are not sure, read the appropriate sections before making your selection.

☒ Life sciences ☐ Behavioural & social sciences ☐ Ecological, evolutionary & environmental sciences

For a reference copy of the document with all sections, see nature.com/documents/nr-reporting-summary-flat.pdf

Life sciences study design

All studies must disclose on these points even when the disclosure is negative.

Sample size	Individuals of community health promotion activity were recruited from 2013 to 2019. A total of 618 subjects aged over 30 years old and have complete and adequate clinical data and serum/urine samples for analysis were included for study.
Data exclusions	Subjects were pregnant, undergoing dialysis therapy, or recipients of renal graft transplantation were excluded.
Replication	An independent cohort (178 subjects) that was used for external validation was collected from individuals who attended outpatient clinics at Chang Gung Memorial Hospital in 2019 and 2020.
Randomization	NA
Blinding	NA

Reporting for specific materials, systems and methods

We require information from authors about some types of materials, experimental systems and methods used in many studies. Here, indicate whether each material, system or method listed is relevant to your study. If you are not sure if a list item applies to your research, read the appropriate section before selecting a response.

Materials & experimental systems

n/a	Involved in the study	
☒	☐	Antibodies
☒	☐	Eukaryotic cell lines
☒	☐	Palaeontology and archaeology
☒	☐	Animals and other organisms
☐	☒	Human research participants
☐	☒	Clinical data
☒	☐	Dual use research of concern

Methods

n/a	Involved in the study	
☒	☐	ChIP-seq
☒	☐	Flow cytometry
☒	☐	MRI-based neuroimaging

Human research participants

Policy information about studies involving human research participants

Population characteristics	The mean age of study population was 63.8±12.9 years old and consisted of 287 males (46.4%). The median eGFR is 83.0 mL/min/1.73 m2. From 618 subjects, 338 subjects (54.7%) were controls, 112 (18.1%) had type 2 DM, 73 (11.8%) had non-diabetic CKD, and 95 subjects (15.4%) had DKD.
Recruitment	A total of 618 subjects aged over 30 years old and have complete and adequate clinical data and serum/urine samples for analysis were included for study. An independent cohort (178 subjects) was also enrolled.
Ethics oversight	This study protocol conforms to the ethical guidelines of the 1975 Declaration of Helsinki and was approved by the Institutional Review Board of Chang Gung Medical Foundation.

Note that full information on the approval of the study protocol must also be provided in the manuscript.

Clinical data

Policy information about clinical studies

All manuscripts should comply with the ICMJE guidelines for publication of clinical research and a completed CONSORT checklist must be included with all submissions.

Clinical trial registration	NCT04839796

Study protocol	Population-based, cross-sectional study
Data collection	Individuals of community health promotion activity of Chang Gung Memorial Hospital at Keelung were recruited from 2013 to 2020.
Outcomes	Occurrence of Diabetes, Chronic Kidney Disease or Diabetic Kidney Disease
